# Supplementary material for: Quality of Life, Clinical, and Patient-Reported Outcomes after Pencil Beam Scanning Proton Therapy Delivered for Intracranial Grade WHO 1–2 Meningioma in Children and Adolescents
Source: Cancers (Basel). 2023 Sep 6;15(18):4447. doi: 10.3390/cancers15184447 (PMC10526222; doi:10.3390/cancers15184447)
Supplement: Supplementary file 1 [file cancers-15-04447-s001.zip › Supplementary material S1. Example of a follow-up questionnaire.pdf]

PAUL SCHERRER INSTITUT

Paul Scherrer Institut
Forschungsstrasse 111
5232 Villigen PSI
Switzerland

+41 56 310 21 11
www.psi.ch

Prof. Damien Charles Weber, MD
Head of Center for Proton Therapy CPT
WPTA/144

direct +41 56 310 35 24
Fax +41 56 310 35 15
protonentherapie@psi.ch

Villigen PSI, DD.MM.YYYY

Name, DOB. DD.MM.YYYY, PXXXXX

Follow Up Questionnaire

Dear Parents,

We would like to inquire about the well-being of your child whom we treated with protons at our institute. Proton therapy delivers a prescribed dose to the cancer target while reducing or avoiding radiation dose to normal tissues. It is also important for us as care-givers to know how your child is doing after the treatment. Feedback from you is critical in assessing the outcome of the treatment as well as its side effects. In most instances, because of the distances involved, it may be inconvenient for you to visit our institute for purposes of follow up examinations. We have, therefore, prepared a questionnaire as a way of regularly interacting with you to learn about the overall progress of your child. We hope you find it comprehensible and easy to complete. We appreciate your efforts to answer the questions as completely as possible.

1. In your opinion, has your child sustained any impairment due to the disease and/or therapy?
   ☐ No ☐ Yes – If so, what are your child’s major issues?
   ____
   ____
2. Is your child participating in any regular education?
   ☐ No ☐ Yes – Which one (kindergarten, primary, occupational, etc.) and at which level?
   ____
   ____
3. Does your child need any special educational support?
   ☐ No ☐ Yes –If so, what kind of support (speech therapy, extra lessons, etc.)?
   ____
   ____

Paul Scherrer Institut
Forschungsstrasse 111
5232 Villigen PSI
Switzerland

+41 56 310 21 11
www.psi.ch

4. Does your child have any difficulty in finding new friends?
   ☐ No ☐ Yes

5. Does he or she have problems in interacting with his or her friends?
   ☐ No ☐ Yes – If so, what kind of problems?
   ____
   ____

6. Does he or she have problems in interacting with members of the family?
   ☐ No ☐ Yes – If so, what kind of problems?
   ____
   ____

7. Does your child have to take any medications regularly?
   ☐ No ☐ Yes – If so, which?
   ____
   ____

8. Does he or she need any help with (or aids for) walking, washing or dressing? Does he or she require any visual or hearing aids?
   ☐ No ☐ Yes – If so, which?
   ____
   ____

9. When did he or she last see the doctor? Please state the doctor's name and hospital or city:
   ____
   ____

10. When and where was the last follow up imaging performed?

CT	Date ________________	Where? ________________ ________________
MRI	Date ________________	Where? ________________ ________________
X-Ray	Date ________________	Where? ________________ ________________

PAUL SCHERRER INSTITUT

Paul Scherrer Institut
Forschungsstrasse 111
5232 Villigen PSI
Switzerland

+41 56 310 21 11
www.psi.ch

PET Scan Date ________________ Where? ______________________ ______________________

Bone scan (Scintigraphy) Date ________________ Where? ______________________ ______________________

Cerebrospinal Fluid Date ________________ Where? ______________________ ______________________

11. What were the results?

____

____

____

12. Has there been any recurrence of the disease; that is, has the tumor grown back?

☐ No ☐ Yes – If so, then where?

☐ Locally, at the original tumor site

☐ If metastases have developed, where are they located?

☐ Lung ☐ Liver ☐ Lymph nodes

☐ Bone ☐ Brain ☐ Other: ________________________________

13. Does your child receive any tumor treatment at the moment? Or has he or she received any tumor treatment during the last year?

☐ No ☐ Yes – If so, which treatment?

☐ Chemotherapy ☐ Surgery ☐ Radiotherapy ☐ Other: ______________________

14. How does the irradiated area look now? (e.g. Skin irritation? Hair growth?)

____

____

15. Weight: ____________________________ Height: __________ Seated height: ____________________

PAUL SCHERRER INSTITUT

Paul Scherrer Institut
Forschungsstrasse 111
5232 Villigen PSI
Switzerland

+41 56 310 21 11
www.psi.ch

16. Please describe any problems your child has been experiencing since the end of proton therapy. Please give the date when the problem started as closely as possible

	Problem	Since when?
a)<br>*b)*	*Examples: Hair loss*<br>*Hormonal imbalance*	*dd.mm.yy*<br>*dd.mm.yy*
1		
2		
3		
4		
5		

17. Is your child currently suffering any other illness, independently of the tumor history?

☐ No ☐ Yes – If so, which?

____

____

18. Is there any other information on your child that you would like to share with us?

____

____

____

19. Which doctor could we contact for further details? (Name, address and telephone number)?

____

____

____

We would appreciate receiving recent follow-up information (reports, images) from you directly, if you have any available

Date: ____________________ Signature: ______________________________

PAUL SCHERRER INSTITUT PSI

Paul Scherrer Institut
Forschungsstrasse 111
5232 Villigen PSI
Switzerland

+41 56 310 21 11
www.psi.ch

We want to thank you and we appreciate your willingness to answer this questionnaire. We would like to send the same questionnaire once a year. Should your contact information change, please let us know. Please feel free to contact us any time via e-mail, letter, fax or telephone

Our address:
Center for Proton Therapy
Paul Scherrer Institut,
Forschungsstrasse 111,
CH-5232 Villigen-PSI, Switzerland

Phone: +41 (0)56 310 35 21
Fax: +41 (0)56 310 35 15
E-mail: ruth.eggspuehler@psi.ch / katharina.sortino@psi.ch

We would like to mention that we would be glad to see you for a follow-up visit, if you should be able come to PSI. We would also appreciate it, if you could periodically send us a few pictures of your child and of the irradiated area. The most convenient method would be to send digital photos as e-mail attachments.

With my personal regards,

Prof. Dr. med. Damien Charles Weber
Head and Chairman, Center for Proton Therapy
